# The Brain Entangled: The Contribution of Neutrophil Extracellular Traps to the Diseases of the Central Nervous System

**DOI:** 10.3390/cells8121477

**Published:** 2019-11-21

**Authors:** Aneta Manda-Handzlik, Urszula Demkow

**Affiliations:** 1 Department of Laboratory Diagnostics and Clinical Immunology of Developmental Age, Medical University of Warsaw, 02-091 Warsaw, Poland; urszula.demkow@litewska.edu.pl; 2 Postgraduate School of Molecular Medicine, Medical University of Warsaw, 02-091 Warsaw, Poland

**Keywords:** neutrophil extracellular traps (NETs), Alzheimer’s disease, multiple sclerosis, ischemic stroke, meningitis, central nervous system, brain, neurons, brain–blood barrier, neutrophils

## Abstract

Under normal conditions, neutrophils are restricted from trafficking into the brain parenchyma and cerebrospinal fluid by the presence of the brain–blood barrier (BBB). Yet, infiltration of the central nervous system (CNS) by neutrophils is a well-known phenomenon in the course of different pathological conditions, e.g., infection, trauma or neurodegeneration. Different studies have shown that neutrophil products, i.e., free oxygen radicals and proteolytic enzymes, play an important role in the pathogenesis of BBB damage. It was recently observed that accumulating granulocytes may release neutrophil extracellular traps (NETs), which damage the BBB and directly injure surrounding neurons. In this review, we discuss the emerging role of NETs in various pathological conditions affecting the CNS.

## 1. Neutrophils in the Central Nervous System (CNS)

Neutrophils, crucial cells of innate immunity, are scarce in the central nervous system (CNS) under normal conditions. They are restricted from trafficking into the brain parenchyma and cerebrospinal fluid (CSF) by the presence of the brain–blood barrier (BBB). Tight junctions between brain endothelial cells ensure barrier integrity and high selectivity [1,2,3]. Yet, the infiltration of the CNS by neutrophils in various pathological conditions, e.g., infection, trauma, brain ischemia, neurodegeneration or autoimmunity, is a well-known phenomenon. Different studies have shown that neutrophil products, i.e., free oxygen radicals and proteolytic enzymes including matrix metalloproteinase 9 (MMP-9), play an important role in the pathogenesis of BBB damage [4,5]. It was recently observed that accumulating granulocytes may also release extracellular web-like structures composed of DNA and proteins called neutrophil extracellular traps (NETs), which damage the BBB and account for subsequent injury of surrounding neurons and other cells of the brain [1,6].

## 2. Neutrophil Extracellular Traps (NETs) in Physiology and Pathology

Although the term “NETs” was coined, and their biological relevance was discovered, by the Zychlinsky group in 2004 [7], it is worth noting that an atypical form of neutrophil death following stimulation with phorbol 12-myristate 13-acetate was identified almost a decade earlier by Takei et al. [8]. Current consensus is that NET release is a highly variable phenomenon, either accompanied by cell survival or ultimately eliciting lytic cell death [9]. Furthermore, the NET backbone can be composed of DNA of nuclear, mitochondrial or both origins [9,10]. An abundance of studies has revealed a broad spectrum of NET targets—including bacteria, parasites, fungi and viruses [11]. Currently, it is widely accepted that the major role of NETs is to entrap and immobilize pathogens, preventing an infection from spreading [7], but much more controversy has arisen around the pathogen-killing properties of NETs [12]. Regardless of the direct effect of NETs on pathogens’ viability, the release of these structures constitutes an efficient antimicrobial strategy. However, it should be underlined that an overabundance of lytic, cytotoxic proteins (including histones, neutrophil elastase (NE) and defensins) and autoantigens (such as DNA, histones, myeloperoxidase (MPO) and proteinase 3) in NETs may have dramatic consequences for the host. The disturbance between NET formation and clearance has thus been implicated in a number of various diseases, both systemic and limited to a certain organ or tissue. For example, excessive formation of NETs contributes to the pathogenesis of psoriasis, systemic lupus erythematosus, diabetes, cystic fibrosis, and cancer [13,14,15,16,17]. As mentioned above, it has been also recognized that NETs can be implicated in brain disorders and other pathological conditions affecting the CNS. In this review, we summarize the current state-of-the-art regarding the role of NETs in neurological pathologies.

## 3. NETs in Ischemic Stroke

**Figure 1:**
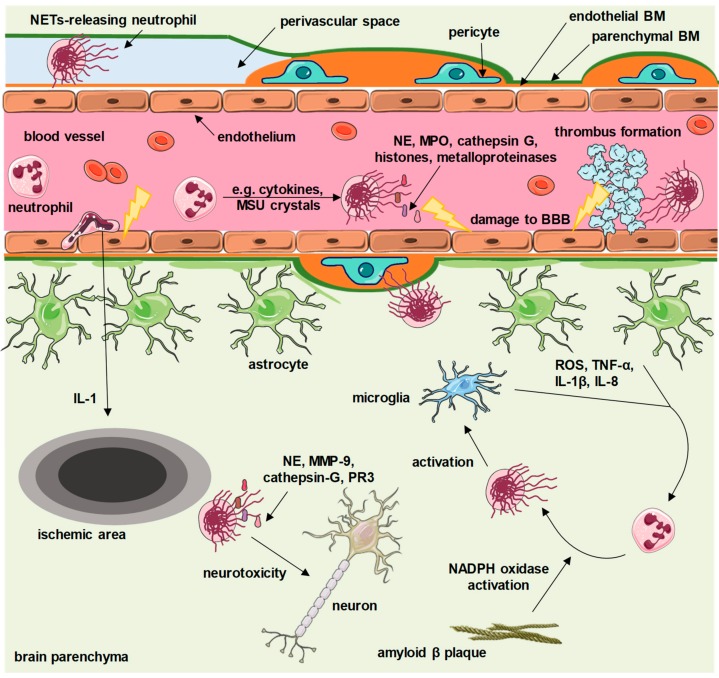
Proposed contribution of neutrophil extracellular traps (NETs) to central nervous system diseases. Depending on the underlying disease, various factors (cytokines, amyloid β plaques, reactive oxygen species (ROS), monosodium urate (MSU) crystals and others) activate granulocytes to release NETs. Intravascular NETs activate the coagulation cascade and enhance formation of thrombi, and also carry cytotoxic proteins that directly damage the brain–blood barrier (BBB). Extravasated granulocytes release NETs within perivascular spaces, as well as within brain parenchyma. NETs exert neurotoxic effects and activate microglia, which further enhances NET release. BM—basement membrane, PR3—proteinase 3, MMP-9—matrix metalloproteinase 9, TNF-α—tumor necrosis factor α, IL—interleukin, NE—neutrophil elastase, MPO—myeloperoxidase, NADPH—the reduced form of nicotinamide adenine dinucleotide phosphate. This figure contains elements available at Servier Medical Art repository, licensed under a Creative Commons Attribution 3.0 Unported License.

Acute brain injury, including ischemic stroke, always initiates local inflammation in the CNS. A key hallmark of neuroinflammation is damage of the BBB and transmigration of immune cells into the brain, leading to neuronal death. Animal studies proved that ischemic areas of the brain are infiltrated by neutrophils within a few hours after the onset of experimental ischemia [18,19,20,21]. Neutrophils are attracted by chemical mediators and damage-associated molecular patterns arising from sterile inflammation invoked by ischemia–reperfusion. Locally produced interleukin (IL)-1 plays a crucial role in this process. IL-1 is responsible for the recruitment and transmigration of neutrophils across damaged BBB [6] (Figure 1). Further activation of these cells in inflamed tissues of the brain is connected with profound changes of their phenotype and release of decondensed DNA threads decorated with extracellular proteases [6]. Accordingly, Perez-Puig et al. described the presence of citrullinated histone 3, a hallmark of NET formation, in the ischemic brain after 24 h ischemia [22]. Positive staining for citrullinated histone 3 was observed in neutrophils expressing typical features of cells undergoing NET release (decondensation of nuclear chromatin) [22]. Neutrophils with characteristic phenotypic changes were found in the lumen of capillaries, in perivascular spaces, in the brain parenchyma nearby blood vessels, and surrounding pericytes, suggesting that NETs might contribute to the damage of the BBB. Additional examination of brain tissue from patients who died from stroke revealed co-localization of MPO and NE in neutrophils found in perivascular spaces [22]. Other authors described the presence of decondensed DNA released from neutrophils in the inflammatory brain lesions of experimental animals [23].

On the one hand, local NETs formation is believed to protect injured brain from further bacterial attack. On the other hand, the inflammatory milieu exerts direct neurotoxic effects. Allen et al. observed that transmigrated neutrophils co-locate with neurons [6]. A number of highly significant associations were found between neuronal loss after ischemic stroke and neutrophil transmigration. Allen et al. [6] showed in vitro that transmigrated neutrophils cultured with neurons for 3 h significantly decreased neuronal viability. This effect was not abrogated by DNase treatment of conditioned medium from transmigrated granulocytes; thus, a decrease of neuronal viability was not attributable to extracellular DNA. Furthermore, the inhibition of neutrophil-derived extracellular proteases associated with NETs significantly decreased neutrophil-mediated neurotoxicity. Interestingly, the key neutrophilic proteases, cathepsin-G, NE, proteinase-3 and MMP-9, seem to collectively attack neurons as shown in experiments when a mixture of their inhibitors, but not any single specific inhibitor, nearly completely reversed the neutrophil-dependent neurotoxic effect [6]. Altogether, these authors identified a novel neuroinflammatory mechanism: the development of rapid neurotoxicity of neutrophils initiated by IL-1–induced cerebrovascular transmigration. Consistently, Allen et al. proved that rapidly developed (30 min) neutrophil-dependent neurotoxicity is mediated by neutrophil-derived proteases released upon degranulation or associated with NETs. Accordingly, these authors proposed a new therapeutic strategy against neuronal death in the course of brain injury, based on blockade of IL-1. Such an approach is believed to protect the brain from NET-dependent neurotoxicity [6].

Further, it was proven that in the course of brain ischemia, web-like structures formed inside and around capillaries enhance thrombus formation (Figure 1). We can hypothesize that histones are crucial thrombogenic components of NETs because it was shown that extracellular histones are potent stimuli for thrombin generation in vitro [24,25]. Examination of thrombi retrieved from the brain circulation of ischemic stroke patients revealed the presence of DNA and citrullinated histone 3 scaffold [22,26,27]. This secondary thrombosis further contributes to the prolongation of the period of ischemia. It is believed that NET formation may be responsible for the no-reflow phenomenon, closing the time window for thrombolytic therapy [22]. This result suggests that intravascular decondensed DNA fibers may play a previously unanticipated role in the resistance to fibrinolytic therapy. Recanalization in patients with acute ischemic stroke is achieved only in less than a half of the patients who receive tissue plasminogen activator (t-PA) within hours of the onset of symptoms. In accord with these observations, t-PA resistance may be attributed to the formation of NET scaffolds enclosing platelets and activating the intrinsic coagulation pathway. Therefore, it has been speculated that NETs promote secondary microthrombosis [22]. It was reported that older thrombi are rich in citrullinated histone 3 and positive for NE, the key hallmarks of NETs, compared to fresh thrombi [28]. These observations may help to devise novel approaches to widen the therapeutic window for fibrinolysis in order to prevent permanent neurological damage of patients with stroke. This conclusion corresponds with the findings that DNase 1 improved the therapeutic efficacy of t-PA [28]. Given the low-cost and safety of DNase 1, which is already FDA-approved for cystic fibrosis therapy, it could, in combination with fibrinolytic therapy, significantly improve the outcome of ischemic stroke patients [28].

Finally NETs are believed to account for the development of stroke-induced systemic immunosuppression [5,29]. Activated granulocytes releasing NETs decrease the T lymphocyte activation threshold in vitro [30]. Even though NETs play a role in the upregulation of CD25 and CD69, and the phosphorylation of the TCR-associated signaling kinase ZAP70, these effects are not associated with the proliferation of CD4+ T cells [30]. Further studies are warranted to discern alternative links between NETs and systemic immunosuppression in the course of ischemic stroke [5].

## 4. Neurodegeneration

Chronic neurodegenerative diseases including Alzheimer’s disease (AD), Parkinson’s disease (PD) and the prion-associated diseases (PAS) are not typically assigned to neuroinflammatory conditions, however some specialists consistently highlight the links between these disorders and the local innate immune response [1]. For example, Zenaro et al. provided evidence that netting neutrophils contribute to the pathogenesis of Alzheimer’s disease (AD) [31]. AD is a neurodegenerative disease characterized by progressive cognitive impairment and memory loss. The most consistent neuropathological feature of an AD brain is the presence of neuritic plaques consisting of amyloid-β and neurofibrillary tangles formed by aggregates of hyperphosphorylated tau-protein. A convincing body of evidence supports the inflammatory background of AD and several subpopulations of blood-derived white blood cells, including neutrophils, have been found in the brains of these patients [32,33,34,35]. Recent studies by Prof. Constantin’s group highlighted that neutrophils transmigrated into brain parenchyma accumulate in close proximity to amyloid-β plaques, as amyloid-β triggers neutrophils’ adhesion to the endothelium and provides a stop signal to crawling cells [31]. Both intravascular adhesion and migration of neutrophils inside the parenchyma in the areas with amyloid-β plaques are controlled by LFA-1 integrin. Strikingly, neutrophils inside the cortical vessels and brain parenchyma released NETs both in transgenic mouse models of AD as well as in individuals with AD. This observation suggested that neuronal injury and damage to the BBB in AD can be at least partially caused by the detrimental effect of NETs on the vessel wall and surrounding tissues. Same authors, in a comprehensive review paper, proposed plausible explanations for the role of NETs in AD pathology [36]. Pietronigro et al. provided evidence for the presence of NETs in the brain capillaries and tissue of AD mice. These results point to the fact that local NET formation may contribute to local BBB damage and loss of neurons in AD [36]. Importantly, endothelial cortical cells in AD subjects are characterized by increased expression of adhesion molecules and production of pro-inflammatory cytokines, such as tumor necrosis factor (TNF-α), IL-8 and IL-1β [37,38]. Adhesion of granulocytes to activated vasculature may stimulate neutrophils to produce reactive oxygen species (ROS) and favour the release of NETs, presumably with the contribution of activated platelets via intercellular adhesion molecule (ICAM)-2 and the lymphocyte function-associated antigen (LFA)-1 interaction. As previously described, intravascular NETs promote thrombosis, which further exacerbates brain microvessel pathology [36]. Furthermore, intravascular NETs can cause direct toxic effects to the endothelium due to the release of proteolytic proteins, such as NE, metalloproteinases (MMPs) and cathepsin G (Figure 1). NE and MMPs are implicated in the disruption of junctional complexes and endothelial cell retraction. NE itself increases endothelial cell permeability, whilst MPO and histones induce endothelial cell death [39,40,41]. Above all, histones have been identified as major NET-associated proteins that induce cell death [24]. Altogether, NETs may represent an important player involved in the loss of BBB integrity. On the other hand, activated glial cells within the parenchyma initiate a vicious cycle, encompassing neutrophils crawling towards amyloid-β plaques. It is suggested that mediators produced by microglial cells and astrocytes, such as ROS, TNFα, IL-1β and IL-8, can easily activate neutrophils to form NETs, which in turn further activate glial cells [36,42]. What is more, amyloid-β activates NADPH oxidase, a key enzyme participating in NET release [43]. Amyloid-β plaques in line with netting neutrophils are postulated to constitute another feedback loop amplifying neuroinflammation [36]. NET constituents can be harmful to neural cells within brain parenchyma, as they proteolytically cleave extracellular matrix proteins, activate inflammasome pathways and the mitochondrial apoptosis pathway [36,44,45,46].

Although NET release seems to provide a sound explanation for many aspects of AD neuroinflammation, the role of NETs in this disease has been recently acknowledged and requires further rooting in experimental data before NETs can be used as a target of AD therapy. It would also be interesting to identify whether NETs induce the generation of autoantibodies in AD and whether they could constitute an AD biomarker [36].

## 5. Autoimmune Diseases

As early as in the very first report on the phenomenon of NET release, it was recognized that NETs expose intracellular antigens and may contribute to the development of autoimmune diseases [7]. Indeed, NETs have been implicated in numerous autoimmune conditions, including systemic autoimmune diseases that may affect the central and peripheral nervous system, as well as neural antigen-specific autoimmunity [11]. For example, elevated levels of circulating NET formation markers were identified in multiple sclerosis (MS). MS is a progressive neurodegenerative disorder with a strong autoinflammatory background, characterized by spatiotemporally separated multifocal demyelination and perivascular inflammation within the CNS [47]. Early studies on circulating NET markers in MS patients argued against a key role of NETs in the pathogenesis of this disease, since only a subset of relapsing remitting MS patients exhibited significant formation of NETs in vivo [48]. Intriguingly, although the level of MPO-DNA complexes did not correlate with disease activity in MS patients, they were more abundant in males than in females, suggesting that the variability in NET release may account for sex-specific differences in MS pathogenesis. NETs were not detected in CSF samples of MS patients, which corresponds with previous reports pointing to the absence of neutrophils within the CNS of MS patients. Yet, it was suggested that cytotoxic components of NETs may contribute to BBB damage in this disease [48] (Figure 1). The putative role of neutrophils and NETs in MS pathogenesis is further supported by data from experimental autoimmune encephalomyelitis (EAE), a model for MS [49,50]. As an example, it has been well documented that NETs activate inflammatory T helper 17 (Th17) cells to produce their signature, neutrophil-recruiting cytokine, interleukin-17 (IL-17) [51,52]. Notably, interfering with neutrophil–IL-17 interactions significantly reduces severity and delays the onset of EAE [53]. Similarly, the IL-17 level is elevated in CSF in MS patients and correlates with neutrophil expansion in CSF as well as with damage to the BBB [54]. Furthermore, EAE is alleviated and BBB function is re-established by depleting NET-associated proteins such as MPO and NE [55,56]. Rodent models of autoimmune CNS disorders provide data that corresponds well with observations in humans. Strikingly, the increased plasma levels of NE in MS patients are associated with clinical disability and lesion burden [57]. All aforementioned premises constitute a solid background for the proposed contribution of NET release to MS pathogenesis, but the functional link between these two phenomena is still far from being elucidated [58]. Accordingly, current studies focus on an in-depth analysis of the role of NETs and their constituents in MS-related inflammation [58,59].

NETs have been also implicated in neuropsychiatric manifestations of SLE. Tay et al. suggested a model of cognitive dysfunction in SLE that assumes that neutrophil activation, transmigration and subsequent intrathecal NET release could be a consequence of cerebral endothelium activation by anti-NR2A/B (anti-*N*-methyl-D-aspartate receptor subunit NR2A/B) autoantibodies. As a consequence, NETs formed within the brain parenchyma promote neuronal cell death, leading to cognitive impairment in SLE patients [60].

Finally, it should be noted that although previous research has focused primarily on NET formation within the CNS, these structures were also identified in histological material from peripheral nerves of patients with other systemic autoimmune diseases [61].

## 6. CNS Infections

CNS infections, such as meningitis or encephalitis, can be caused by various pathogens including bacteria, viruses, parasites and fungi. These devastating conditions, resulting from a local failure of the immune response mechanism, may ultimately lead to irreversible brain damage. Although the contribution of neutrophils to brain infections has been investigated for decades, the discovery of NETs provided new insights in the field by identification of a new player with a previously unanticipated role in these disorders. Nevertheless, a clear role of NETs in the infected CSF compartment and in brain tissue is still far from being elucidated. Lumbar puncture followed by the examination of CSF from patients with bacterial meningitis reveals massive transmigration of neutrophils across the BBB [62]. Other authors observed intensive infiltration of neutrophils in leptomeningitis and intraparenchymal vasculitis [63,64,65]. Recent literature reports that NETs are formed in the CSF of patients with pneumococcal meningitis, but not in viral meningitis, CNS borreliosis and subarachnoid haemorrhage [65,66]. In vitro culture of human neutrophils with bacteria isolated from meningitis patients (*S. pneumoniae*, *N. meningitidis*, *L. monocytogenes*, *S. aureus*, *E. coli*, *A. baumanii*, *S. oralis*, *S. capitis* and *S. epidermidis*) revealed that all except *L. monocytogenes* induced NETs [66]. Shotgun proteomic analysis of the CSF from patients with meningitis confirmed the presence of NET-related proteins, such as MPO, NE, proteinase-3 (PR3), cathelicidin LL-37, MMP-9, heparin binding protein (HBP), neutrophil gelatinase-associated lipocalin (NGAL), and histones [66]. Mohanty et al. also detected the presence of NETs in the CSF from rats with pneumococcal meningitis [66]. In order to shed light on the role of NETs in the pathogenesis of meningitis, these authors performed a set of experiments using a rat meningitis and an in vitro model, attempting to degrade NETs with DNase I. They discovered that DNase I significantly cleared bacteria in affected organs (lungs, brain, spleen) and decreased bacterial viability in the presence of neutrophils in vitro. The eradication of bacteria from the brain of DNase-treated rats correlated with the decrease of IL-1β levels. This effect was abrogated by inhibitors of phagocytosis, NADPH oxidase and MPO, confirming the role of phagocytosis and oxidative stress as bactericidal mechanisms in meningitis. Accordingly, NETs participate in the detrimental response to *S. pneumonia* infection, promoting pneumococcal survival in the brain by protecting them from phagocytosis and killing by bactericidal factors. Previously Beiter et al. also observed that pneumococci are entrapped but not killed by NETs [67]. These observations correspond with the findings of the clinical study performed by Tillet et al., who noted a 26% decline in mortality from pneumococcal meningitis after addition of DNase to penicillin therapy [68]. Studies detailing the NET-evading mechanisms proved that pneumococci can produce nucleases or modify the cell surface to avoid NET-mediated killing and to further disseminate to other organs [67,69,70]. Another strain of bacteria with the ability to survive in NETs is methicillin-resistant *S. aureus* [71]. Studies by Mohanty et al. [66] highlighted the complex interplay between various inflammatory mechanisms, including NETs, during pneumococcal meningitis.

In the course of bacterial sepsis, the presence of NETs has been demonstrated in the blood. As described previously, circulating NETs activate the coagulation system, increasing viscosity and changing the rheological properties of the blood [72]. Accordingly, changes in CSF hydrodynamics, as a consequence of NET generation in the CSF compartment, may hinder CSF circulation leading to the development of oedema and increased intracranial pressure [73].

Further study addressing the major role of NETs and NET-degrading DNAses in meningitis was undertaken by de Buhr et al. [65]. These authors demonstrated the presence of NETs in *S. suis* meningitis despite the activity of both host and bacterial DNases in the CSF of infected piglets. Furthermore, de Buhr et al. used an in vitro model of *S. suis*-infected human choroid plexus epithelial cells to examine NET formation and degradation. They found that transmigrated granulocytes vigorously released NETs in the “CSF compartment” to entrap *S. suis* bacteria. These web-like structures were not degraded by two pathogen DNases: SsnA and EndAsuis, previously shown to degrade NETs in vitro [74,75]. In line with these observations, the authors identified two host antimicrobial proteins: human and porcine cathelicidins (respectively, LL-37 and PR-39), which may stabilize NETs and protect them from degradation.

Like many other mechanisms of the immune response, NETs can be both detrimental and protective. Aforementioned studies by de Buhr et al. and Mohanty et al. highlight the diverging effects of NET release in CNS [65,66]. Remarkably, some pathogens become entrapped in NETs to prevent an infection from spreading [65], while others benefit from spatial support provided by these three-dimensional structures and easily become disseminated [66].

Besides meningitis, NETs exert a detrimental effect on BBB integrity and toxicity towards neurons in other infectious diseases affecting CNS. For example, NETs have been proposed to contribute to the loss of BBB integrity in the course of cerebral malaria [76]. Infected red blood cells rupture and release precipitated uric acid (monosodium urate, MSU) crystals, which constitute a potent inducer of NETs [77,78] (Figure 1). Importantly, circulating NETs entrapping parasites were identified in the vasculature of children infected with *Plasmodium falciparum* [79]. As mentioned before, NET fibers may provide a scaffold for the activation of the coagulation cascade, which on one hand protects endothelial cells from damage by MSU crystals, but on the other hand reduces blood flow to end organs, or, in the worst-case scenario, completely abrogates perfusion or triggers disseminated intravascular coagulation. Concurrent processes of NET release and thrombus formation result in the production of inflammatory factors, which compromise BBB integrity and lead to the development of cerebral malaria, being the most severe neurological complication of malaria infection [76,80].

## 7. Peripheral Diseases with Infiltration of Central Nervous System by Neutrophils

Several lines of evidence have indicated that peripheral (e.g., cancer outside the CNS) or systemic diseases, such as sepsis, may result in neuroinflammation and accumulation of myeloid cells in the CNS [81,82,83,84]. Furthermore, diseases primary affecting organs other than the brain, might present with neurological manifestations. For example, cancer patients with a tumor localized outside the CNS, are often characterized by fatigue, tremors, gait disorders, visual disturbances, motor and sensory deficits, as well as cognitive dysfunction, developing prior to cancer diagnosis/therapy [84,85,86]. Yet, exact mechanisms of CNS-mediated cancer symptoms have not been well understood so far. The importance of the aforementioned observations has been recently underscored by Burfeind et al., who identified neutrophils as key role players promoting neuroinflammation and the occurrence of neurological symptoms in a murine model of pancreatic ductal adenocarcinoma (PDAC) [84]. The authors demonstrated that myeloid cells (with neutrophils as the predominant type of infiltrating cells) were recruited to the brain early in the course of malignancy and this was mediated by the chemokine receptor type 2 (CCR2)/C-C motif chemokine ligand 2 (CCL2) axis. Granulocytes accumulated in the velum interpositum, meninges adjacent to regions regulating behavior, appetite and body composition, degranulated and released NETs identified as threads co-locating MPO and citrullinated histone 3. Furthermore, disturbance of CCR2–CCL2 signaling attenuated neutrophil accumulation and alleviated CNS-driven disorders, such as anorexia and muscle catabolism, observed in mice inoculated with cancer cells [84]. Although the exact role of NET release as a mechanism contributing to neurological disorders in PDAC has not been investigated, future studies are warranted to shed new light on these issues.

## 8. May NETs Play a Role in the Development of Brain Tumors?

The CNS is a frequent site for different kinds of primary tumors and metastases from distant organs (i.e., lung cancer, breast cancer and melanoma). The most common primary CNS malignancies encompass a wide spectrum of over 150 histologically, molecularly and clinically distinct conditions, including gliomas and non-glial tumors (meningiomas, medulloblastomas) [87]. The brain tumor microenvironment (TME), crucial for the growth and progression of a tumor, is composed of extracellular matrix components, various mediators and cells: endothelial cells, pericytes, fibroblasts and immune cells including neutrophils [88]. TME is a critical regulator of cancer progression and the response to therapy, thus it may exert a pro-or anti-tumorigenic effect [89]. The observations of both human and animal brain tumors showed that neutrophils are crucial players in TME. These cells are able to cross the BBB and brain-tumor barrier (BTB) to infiltrate the tumor [90,91]. They are attracted to the TME by numerous chemotactic factors, such as IL-8, TNF and CCL2, released by malignant or surrounding cells [92]. Tumor-associated neutrophils (TANs) in the brain release mediators that further attract new populations of neutrophils. These cells have been shown to become activated and to modulate tumor cell motility, migration and invasion [88,93]. For example, TANs show enhanced NADPH (reduced nicotinamide adenine dinucleotide phosphate) oxidase activity, which leads to the production of ROS, especially hydrogen peroxide, which are cytotoxic to tumor cells [94]. Notably, depending on the environmental setting, NET generation may sharply rely on the function of active NADPH oxidase [95,96]. Even though there is no direct experimental evidence on the link between NETs and CNS malignancies, consistent with numerous studies highlighting the prominent role of NETs in tumor growth and metastasis formation in all kind of malignancies, it can be anticipated that NETs in the brain mediate the same effects. Some preliminary evidence indicates that NET-related proteins such as elastase, proteinase-3 and cathepsin G enable invasion of brain tumors by degradation of the extracellular matrix structures [97,98]. Furthermore, the presence of extracellular citrullinated H3 was confirmed in the circulation of cancer patients, including 129 patients with brain tumors. In the case of many other tumors, it was proved that NETs prepare the metastatic niche by entrapping circulating tumor cells [99]. Additionally, NETs promote adhesion of tumor cells to distant organ sites and the presence of NETs in the capillaries of the liver enables the formation of micrometastases in this organ [100,101]. These lines of evidence can indirectly support the hypothesis that NETs may confer similar effects for both primary brain tumors and metastases. A number of highly significant associations were found between neutrophils and the response to therapy of brain malignancies [91,102]. Furthermore, numerous reports point to the negative prognostic value of neutrophil presence and their participation in neuroinflammation in the milieu of brain tumors [91,93,102,103,104,105]. As noted above, current evidence only indirectly points to the participation of NETs in the biology of primary and secondary brain tumors, and we must acknowledge that this issue has not been thoroughly studied yet. Accordingly, further intensive studies are warranted in order to explore this issue and to open new possibilities for therapeutic interventions in those detrimental conditions.

## 9. Conclusions

An increasing body of evidence suggests that NET formation in the CNS might be a common phenomenon, occurring in many brain disorders of various origin. In the present paper, we aimed to describe the role of NETs across a variety of brain disorders driven by a complex of interacting mechanisms. We consider NETs as an element of disease-specific mechanisms; however, in parallel, we have revealed the underlying unity of mechanisms across different brain diseases. A universal, over-arching machinery gives rise to the disruption of BBB integrity and the increase of its permeability, microcirculatory disturbances, vascular leakage, thrombosis, release of proinflammatory cytokines, oxidative stress, neuronal injury and death as well as neuroinflammation. Netting neutrophils have the capacity to actively participate in these cellular and molecular cascades, leading to inflammation and cell death by releasing metalloproteinases, proteases, cytokines, extracellular histones, DNA and ROS. The essentially similar pathogenic mechanisms can diversify over time depending on the initial insult, nature and location of the injury. Further efforts will hopefully address the question of whether this newly recognized relationship between the CNS disorders and NET formation can influence future diagnostic strategies and open novel therapeutic avenues for individuals suffering from the aforementioned conditions.
